# Plain Language Summary of Publication: Design of the Global Prospective Hemolytic Disease of the Fetus and Newborn Registry (GERANIUM)

**DOI:** 10.1055/a-2815-9970

**Published:** 2026-03-20

**Authors:** May Lee Tjoa, Ashley Orillion, Raymond Mankoski, Nida Imran, Carol Mao, Alexis Krumme, Sylvie Van Hoorde, Blanca Linares-Rivas Rico, Yosuke Komatsu

**Affiliations:** 1 Johnson & Johnson, Cambridge, Massachusetts, United States; 2 Johnson & Johnson, Horsham, Pennsylvania, United States; 3 The NemetzGroup, Boston, Massachusetts, United States; 4 Johnson & Johnson, Beerse, Belgium; 5 Johnson & Johnson, Madrid, Spain; 6 Johnson & Johnson, Spring House, Pennsylvania, United States

**Keywords:** hemolytic disease of the fetus and newborn, study design, registry, real-world data, pregnancy outcomes

## Abstract

This article is a plain language summary of publication (PLSP) of the following article: “Study Design of the Global Prospective Hemolytic Disease of the Fetus and Newborn Registry (GERANIUM)” by Tjoa et al published by the *American Journal of Perinatology* on March 20, 2026. This PLSP describes the design of the GERANIUM registry, which will collect data to help researchers understand how doctors manage hemolytic disease of the fetus and newborn (HDFN) in clinical settings. Researchers will also study outcomes related to the health and well-being of pregnant participants affected by HDFN, their children, and their families over time. This PLSP aims to help the general public, including those affected by HDFN, and health care professionals understand the design of the GERANIUM registry.

## Synopsis

### What Is This Summary about?

This is a summary of a publication describing the design of the Global Prospective Hemolytic Disease of the Fetus and Newborn Registry (GERANIUM) in pregnant participants at risk for hemolytic disease of the fetus and newborn (HDFN). The study will collect real-world information on how HDFN-affected pregnancies are currently treated and the health effects for both the pregnant individual and their child during pregnancy and after birth for up to 2 years.

### What Happens in the Study?

The GERANIUM registry is a non-interventional study, meaning that participants will receive care recommended by their doctor. The study sponsor will not provide a particular medicine to test or require any specific study visits or procedures. The GERANIUM registry aims to include around 175 pregnant participants who are at risk of developing HDFN during their pregnancy. Participants will be recruited by approximately 21 medical centers in multiple countries specializing in the diagnosis and treatment of HDFN. At enrollment, previous pregnancy data will be collected from participants' medical records. Doctors will collect relevant information about outcomes of interest during the current pregnancy and after delivery for the pregnant participants and their children for up to 2 years. Questionnaires will also be given to understand how HDFN affects the quality of life for pregnant participants, their families, and their infants/children.

### What Key Results Will the Study Provide?

Researchers will use the data they collect to learn how HDFN is currently treated and what the outcomes of treatment are. The main assessment will determine the number of pregnancies without the loss of a fetus or baby, the need for intrauterine transfusion (IUT; a blood transfusion given to the baby before birth), or the development of fetal hydrops (a serious condition before birth where too much fluid builds up in the baby's body). Researchers will also assess other data related to the treatments received, including pregnancy outcomes; the babies' birth characteristics (e.g., weight and bilirubin levels), health, growth, and development after birth; quality of life; use of medical services, such as hospital, emergency room, or doctor's office visits; and biologic risk factors measured in optional blood tests. These data will help researchers understand how HDFN affects the health and well-being of the pregnant participants, their babies/children, as well as their families/caregivers.

### Who Is This Summary for?

This summary will help members of the public, especially individuals and families affected by HDFN, understand the goals of the GERANIUM registry and how it is being conducted. It may also be helpful for health care professionals. More detailed information and references can be found in the original article. 1 The link to the original article can be found at the end of this summary.

### Who Sponsored the Study?

This study is sponsored by Johnson & Johnson.

## Content

### What Is Hemolytic Disease of the Fetus and Newborn?

**Fig. 1:**
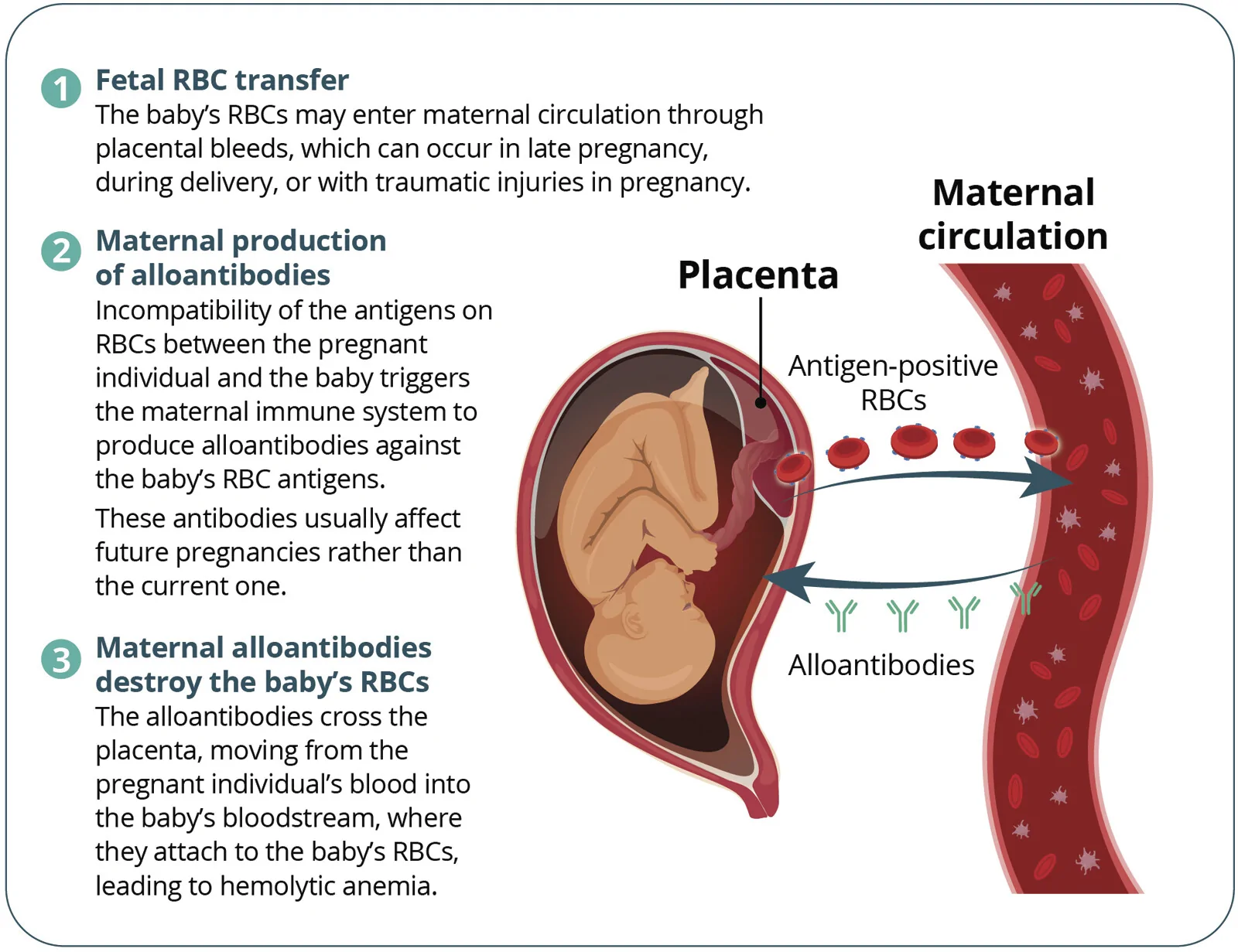
Development of HDFN. HDFN, hemolytic disease of the fetus and newborn; RBC, red blood cell.

HDFN is a rare condition that can occur during pregnancy when proteins (called antigens) found on the surface of the baby's red blood cells (RBCs) are different from those on the pregnant individual's RBCs ( Fig. 1 ). This mismatch causes the immune system of the pregnant individual to see the antigens as foreign material. In response, the body makes antibodies, which help the immune system to find and remove foreign material to avoid getting sick. In HDFN, harmful antibodies, called alloantibodies, are made. These alloantibodies can enter the baby's bloodstream from the pregnant individual by crossing the placenta. The alloantibodies attach to the baby's RBCs, causing them to be destroyed. As a result, the baby can develop hemolytic anemia, a condition where there are not enough healthy RBCs to carry oxygen around the body.

Severe HDFN is uncommon, but if it has happened in a previous pregnancy, the chance of it happening again is much higher in future pregnancies. In these cases, HDFN often starts earlier because the pregnant individual's immune system has already learned to recognize the incompatible antigens from the baby. Severe HDFN can cause serious problems before birth, such as fetal hydrops (when excess fluid builds up in the baby's body) and preterm delivery. After birth, affected babies may develop high levels of bilirubin, a substance the body makes when RBCs are destroyed, in the blood (hyperbilirubinemia). Brain damage can be caused by very high levels of bilirubin (kernicterus). In some cases, the newborn may have ongoing anemia that could require treatments like blood transfusions or plasma exchange. If severe anemia is not treated properly, it can be life-threatening for the baby.

### What Treatments Are Available for Pregnancies at Risk of Hemolytic Disease of the Fetus and Newborn?

**Fig. 2:**
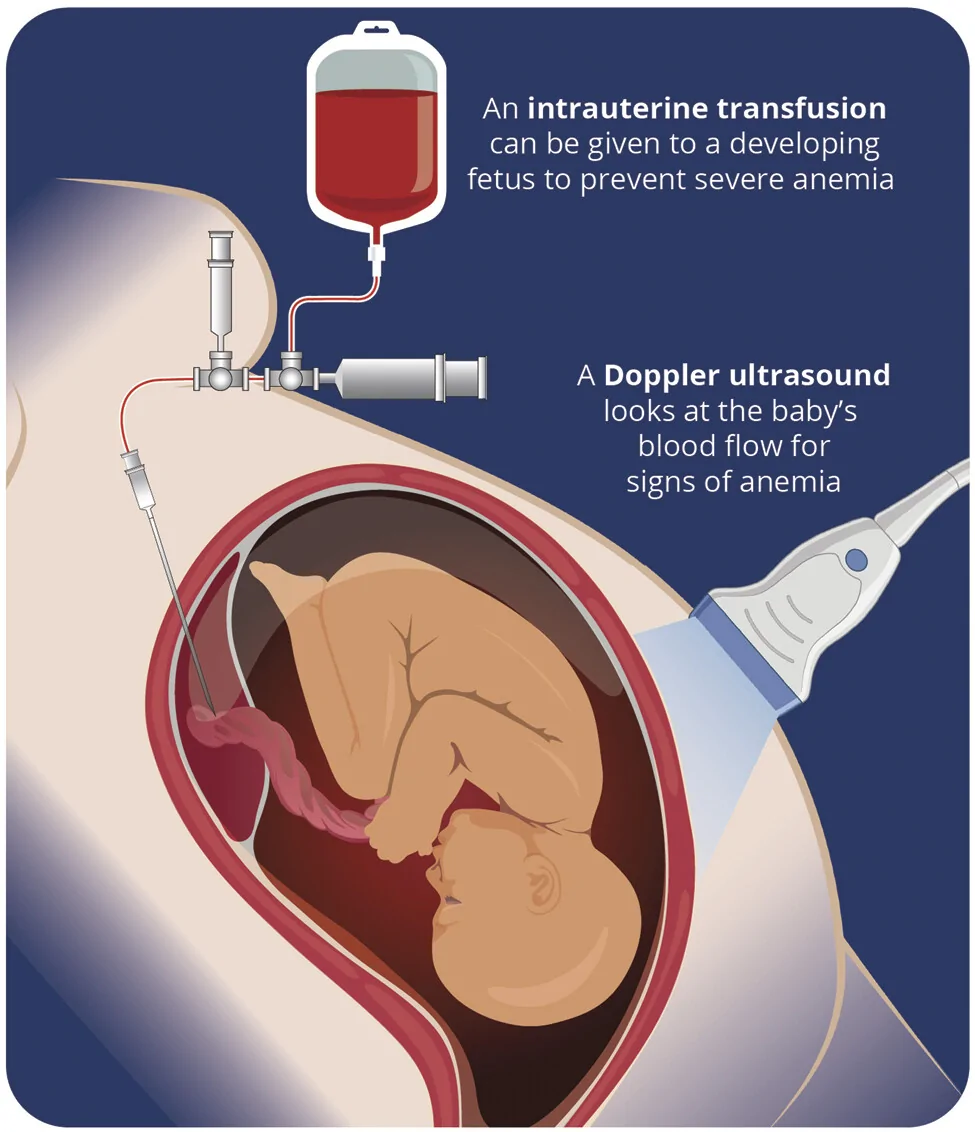
Current management of pregnancies at risk of HDFN. HDFN, hemolytic disease of the fetus and newborn.

During pregnancy, doctors closely monitor babies at risk of HDFN to check for signs of anemia using a special noninvasive technique called a middle cerebral artery Doppler ultrasound ( Fig. 2 ). This examines changes in the baby's blood flow, which may provide signs of anemia. If severe anemia is suspected, doctors take a small sample of the baby's blood from the umbilical cord (cordocentesis) to confirm the diagnosis. If confirmed, the current standard-of-care treatment is to perform a specialized procedure called an IUT to give the baby a blood transfusion before birth. However, IUTs are invasive and carry risks for both the pregnant individual and the baby. These risks may include emergency cesarean delivery, premature birth, infection, pregnancy loss, and increased immune reaction against the baby's RBCs. In some medical centers, doctors may use treatments like intravenous immunoglobulin (IVIg; a treatment made from purified antibodies from donated adult blood) and plasma exchange (a medical procedure used to remove some antibodies from the pregnant individual's blood) to delay the development of anemia and the need for IUTs. However, there is limited evidence on how well IVIg works, and many babies still need IUTs despite these treatments.

### How Can Researchers Learn More about How Doctors Treat a Condition Like Hemolytic Disease of the Fetus and Newborn?

Because treatment of HDFN has evolved in recent years, researchers would like to learn more about how doctors currently manage the disease and what the outcomes are in real-world clinical settings. A “registry” can be used to collect this type of information. It is not a clinical trial, so no new treatments are being tested. There are no required study visits, clinical examinations, or medical procedures. Each participant receives the care recommended by their doctor.

### What Is the GERANIUM Registry?

The GERANIUM registry will collect real-world information on the management of HDFN and related outcomes from pregnant individuals, babies/children, and families/caregivers during pregnancy and for up to 2 years after delivery. By following participating families over time, this registry will help researchers and doctors understand the course of HDFN, how it is treated in different regions, and how it affects babies' health and development. This knowledge can guide best practices, improve care, and ultimately help families and health care providers make more informed decisions in the future.

### Where Will the Study Take Place, and Who Will Take Part in the Study?

**Fig. 3:**
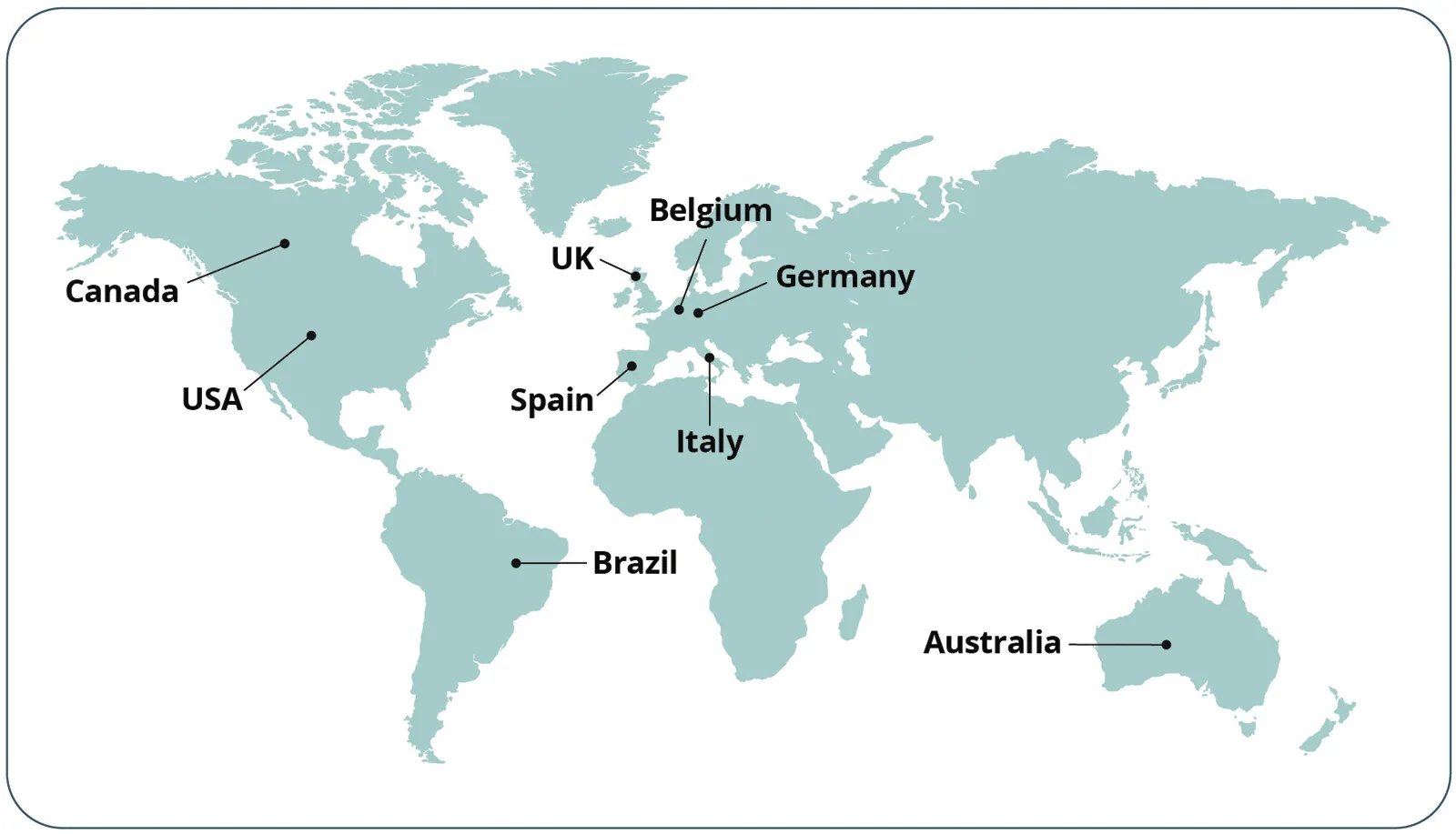
Location of participating centers in the GERANIUM registry. GERANIUM, Global Prospective Hemolytic Disease of the Fetus and Newborn Registry. Formore information on the GERANIUM registry study sites, please visit *https://clinicaltrials.gov/study/NCT07194070* .

**Fig. 4:**
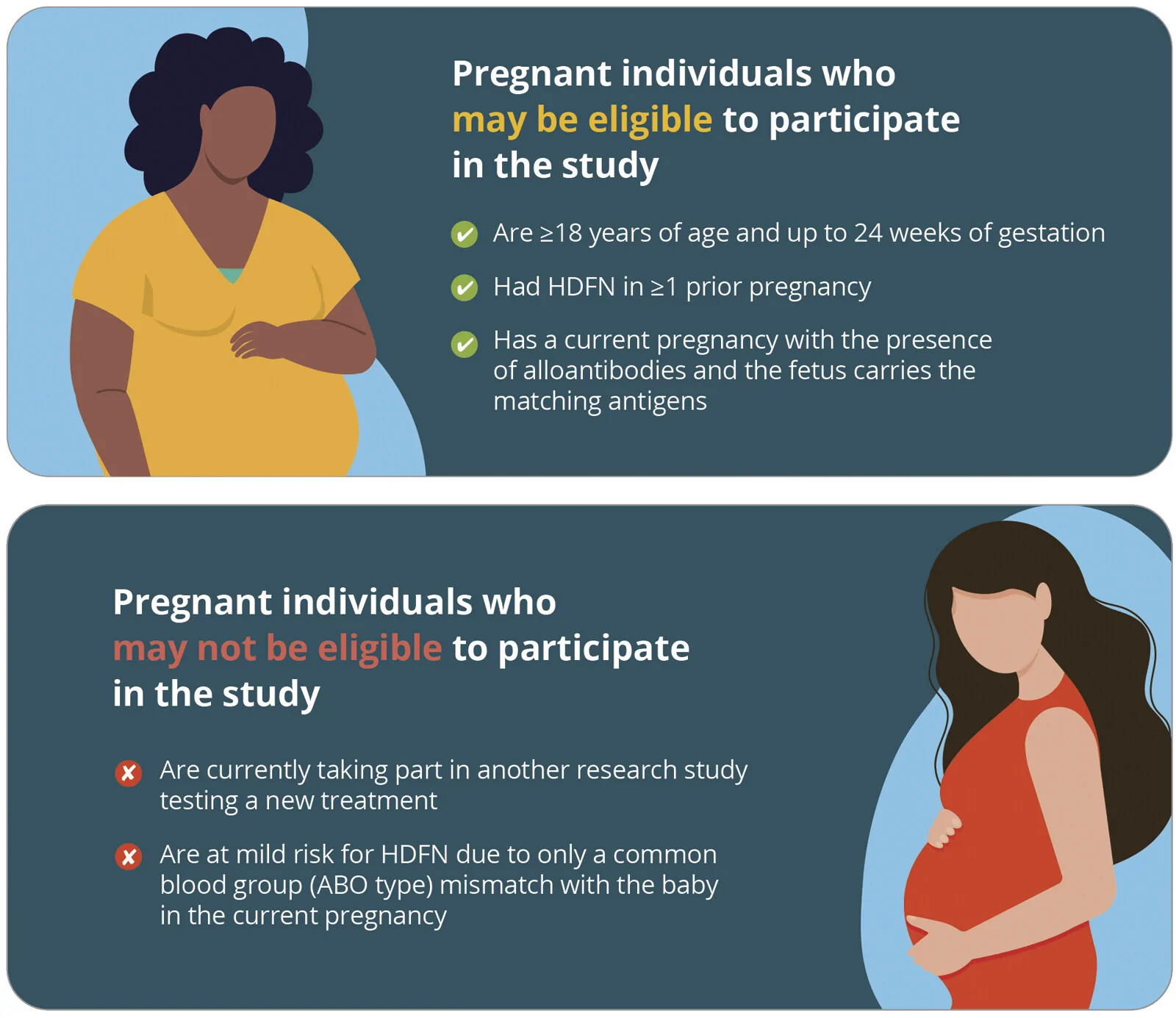
Select eligibility criteria for the GERANIUM registry. HDFN, hemolytic disease of the fetus and newborn; GERANIUM, Global Prospective Hemolytic Disease of the Fetus and Newborn Registry. Complete eligibility criteria can be found in the original article or by visiting *https://clinicaltrials.gov/study/NCT07194070* .

The GERANIUM registry will recruit approximately 175 pregnant participants from specialized medical centers across approximately 21 sites in North America, Latin America, Europe, and the Asia Pacific ( Fig. 3 ). To be eligible, pregnant participants should have evidence that the current pregnancy is producing alloantibodies corresponding to their babies' antigens, and they should have had a previous pregnancy affected by HDFN. Eligible participants can be enrolled at up to 24 weeks of gestation ( Fig. 4 ).

### What Outcomes Will Be Assessed from the Data Collected?

**Fig. 5:**
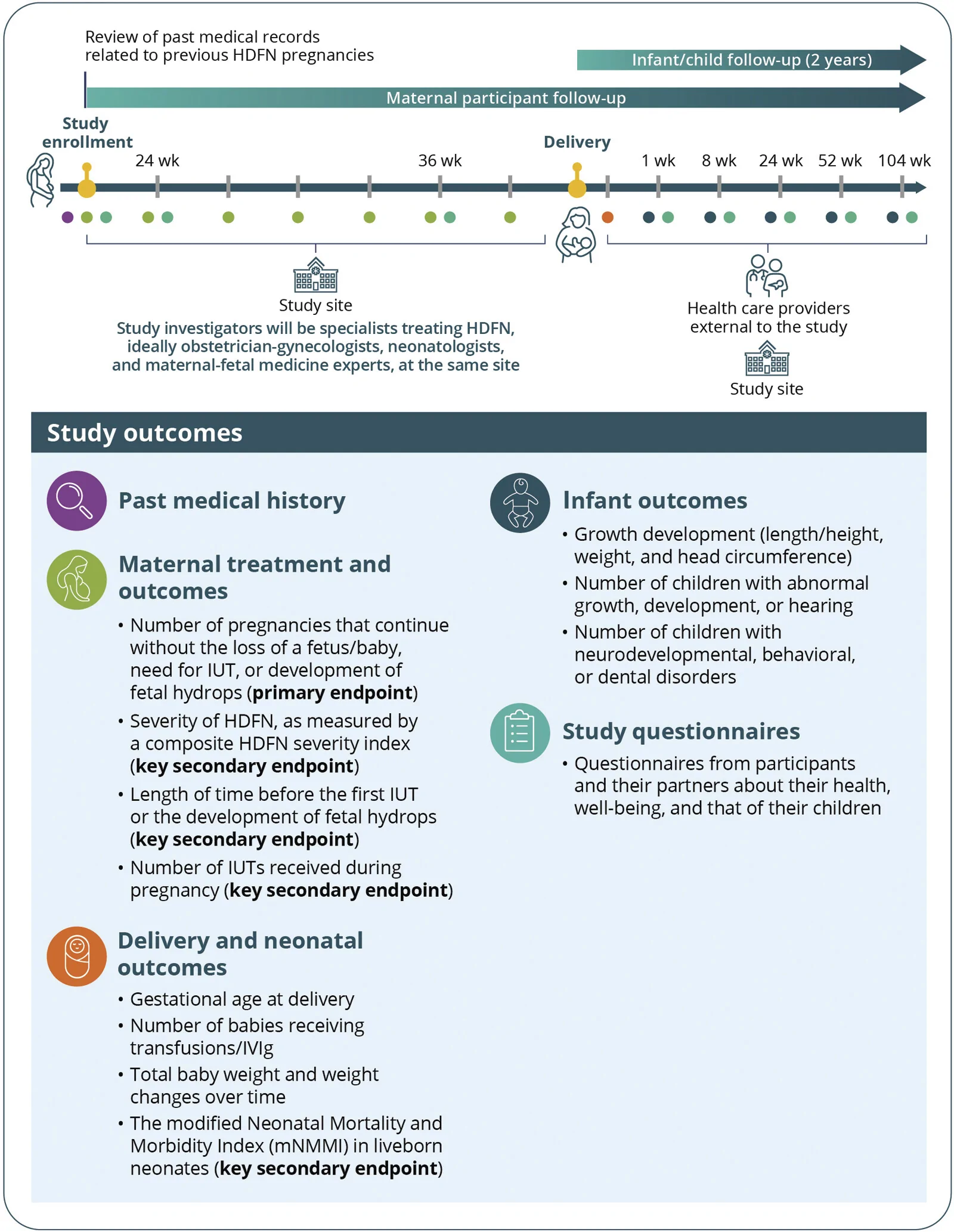
Study design and selected outcome assessments in the GERANIUM registry. GERANIUM, Global Prospective Hemolytic Disease of the Fetus and Newborn Registry; IUT, intrauterine transfusion; IVIg, intravenous immunoglobulin. Complete list of assessments can be found in the original article and at *https://clinicaltrials.gov/study/NCT07194070* .

The GERANIUM registry will collect information prospectively, meaning the researchers plan to study outcomes that have not yet happened. They will use medical records from both the current pregnancy and, if available, from any earlier affected pregnancies ( Fig. 5 ). They will also give optional questionnaires to gather data from pregnant individuals during their pregnancy and for up to 2 years after delivery. Data about the children from their current pregnancy will also be collected over the first 2 years of life. Blood samples may also be obtained from pregnant participants during a routine pregnancy visit to measure the levels of health markers in the blood if they choose to participate in this optional part of the study.

The information obtained will be used to measure several outcomes ( Fig. 5 ). The main outcome, or “primary endpoint,” will evaluate the number of pregnancies that continue without the loss of a fetus/baby, the need for IUT, or the development of fetal hydrops up to 4 weeks after birth or until the baby reaches 1 week after their due date (whichever comes later).

During pregnancy, details about HDFN diagnosis (including severity), other pregnancy complications, fetal assessments, treatments received (e.g., IUT, IVIg, and plasma exchange), and any side effects of treatment will be gathered. At birth, the information collected will include delivery details, along with the baby's characteristics, outcomes, complications, neurodevelopmental assessments, and laboratory results. If the baby needs to stay in the hospital, information collected will include any treatments, outcomes, laboratory evaluations, growth and development assessments, and the use of medical services, such as the length of stay in the neonatal intensive care unit. Following hospital discharge and for up to 2 years after delivery, measures of the baby's growth, side effects of any treatments, other medical conditions, use of medical services (like doctor and emergency room visits), and neurodevelopment will be studied. Additionally, during and after pregnancy, voluntary questionnaires will be given to both pregnant participants and their relatives to evaluate the impact of HDFN on the health and well-being of participants, as well as their children, family members, partners, and caregivers. Researchers will also obtain data on visits to health care facilities, including hospitals, emergency rooms, or doctors' offices, throughout the study.

### What Do Researchers Hope to Learn from the GERANIUM Registry?

The GERANIUM registry will provide new information about how HDFN is treated in everyday clinical practice across different settings. Importantly, data from the pregnant participants and their children will be connected, so researchers will have a clearer picture of how HDFN affects families throughout pregnancy and in the early years of infancy/childhood. By showing how HDFN is managed today and the outcomes for pregnant individuals, their babies, and their families, the GERANIUM registry may help researchers and doctors find ways to improve patient care, help shape future research, and provide support to families affected by HDFN worldwide.

### Where Can Readers Find More Information on This Study?

#### You Can Read the Original Article Here:

The complete bibliography of the GERANIUM registry is presented in the reference. 1

The full name of the GERANIUM registry is “A Study on Hemolytic Disease of the Fetus and Newborn (HDFN) Through Global Registry.” The GERANIUM registry started on July 10, 2025, and is expected to end in 2030.

You can read more about the GERANIUM registry by visiting *https://clinicaltrials.gov/study/NCT07194070* . If you are interested in participating in the study or have questions about the study, please visit *https://clinicaltrials.jnj.com/en/study-detail/NCT07194070* . More information on clinical studies in general can be found at *https://www.clinicaltrials.gov/ct2/about-studies/learn* .
