# Supplementary material for: Plain Language Summary of Publication: Design of the Global Prospective Hemolytic Disease of the Fetus and Newborn Registry (GERANIUM)
Source: Am J Perinatol. 2026 Mar 20;43(13):1828–33. doi: 10.1055/a-2815-9970 (PMC13581422; doi:10.1055/a-2815-9970)
Supplement: Supplementary file 1 [file 10-1055-a-2815-9970_29603279.pdf]

A registry study will gather information about the current management of and outcomes for hemolytic disease of the fetus and newborn (HDFN).

What is HDFN?

- HDFN can develop during pregnancy when the **antigens found on red blood cells (RBCs) of a pregnant individual and their developing fetus do not match**. In response, the pregnant individual's immune system produces **harmful antibodies called alloantibodies**. These alloantibodies can cross the placenta where they attack the baby's RBCs, causing hemolytic anemia. Typically, this process affects future pregnancies rather than the current one
- Severe HDFN is uncommon, but severity is likely to increase with each pregnancy for individuals who have previously had an affected pregnancy
- HDFN may lead to serious problems in the developing fetus or baby, including fetal hydrops, high levels of bilirubin, preterm delivery, anemia, and death

Antigens are proteins found on the surface of RBCs that can trigger the immune system to produce antibodies.

Antibodies are proteins made by the immune system to protect against foreign antigens.

Alloantibodies are a type of antibody that is made by the pregnant individual's body in response to antigens found on their developing fetus.

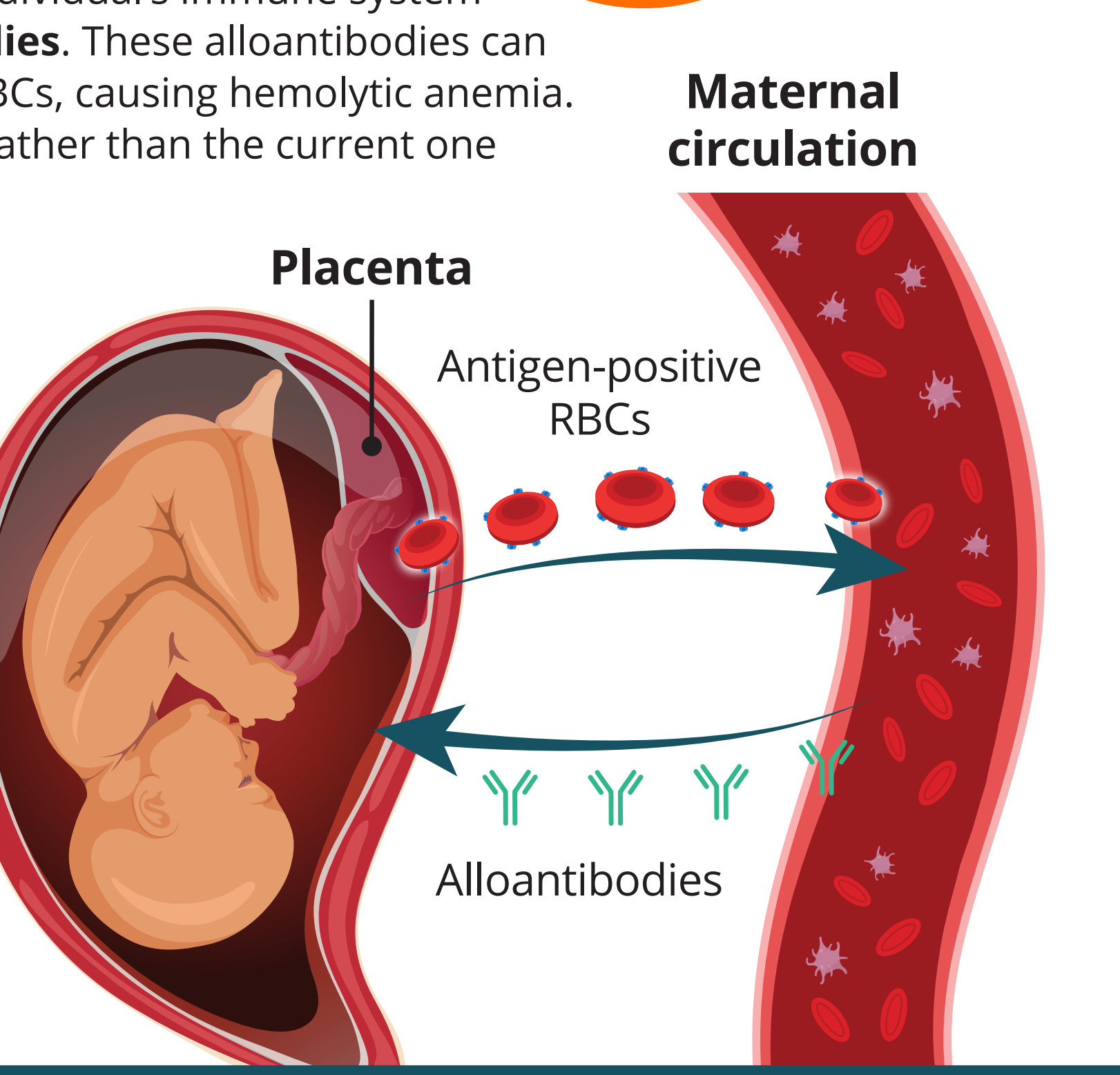

How is HDFN treated now?

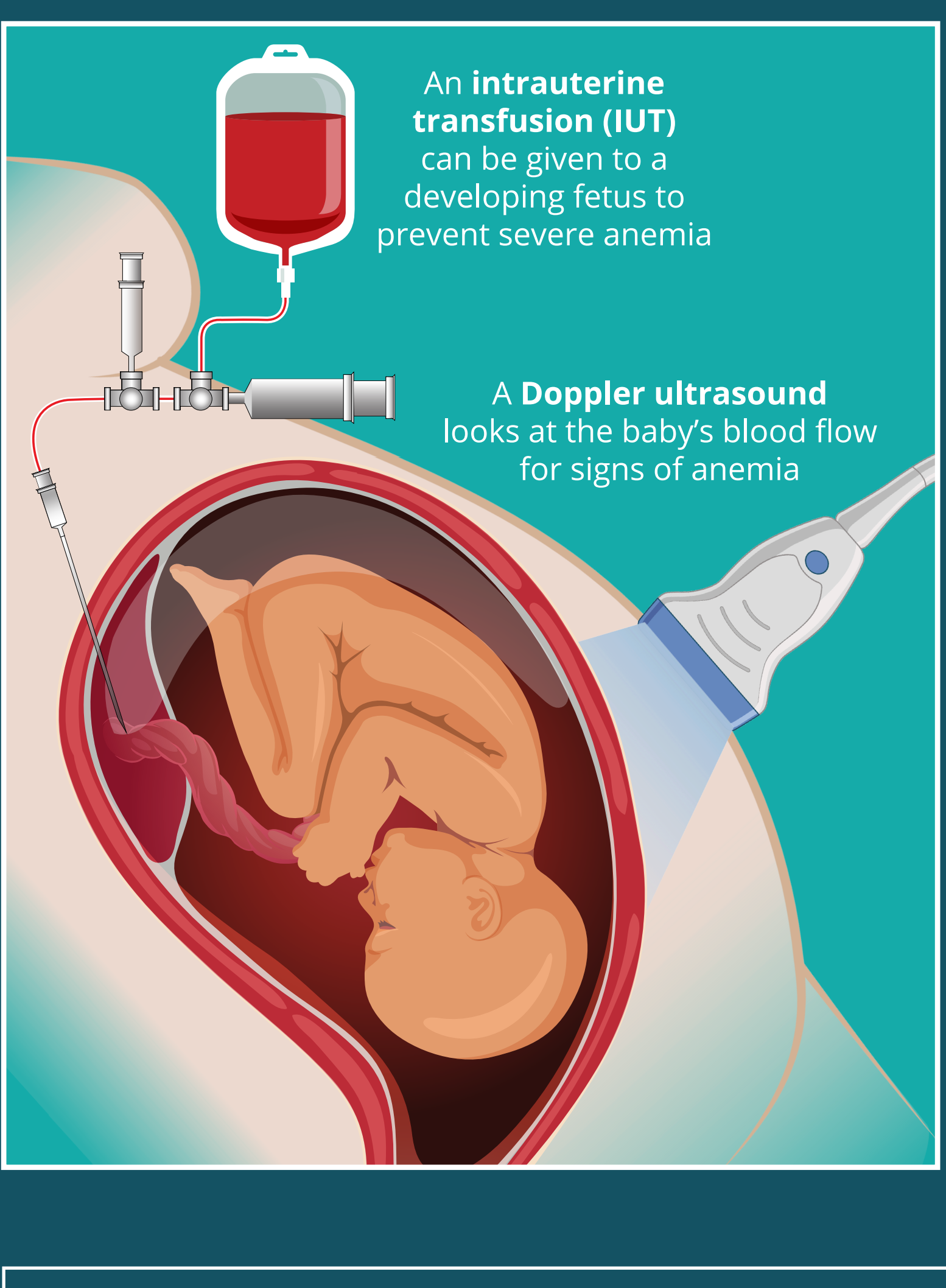

- During pregnancy, doctors closely monitor babies at risk of HDFN using a **Doppler ultrasound of the middle cerebral artery (MCA)**, checking for signs of anemia
- If anemia is confirmed, doctors may perform a blood transfusion on the fetus called an **IUT**
- However, IUTs are invasive and carry risks for both the pregnant individual and the baby, including emergency cesarean delivery, premature birth, infection, and pregnancy loss
- Some doctors may treat with **intravenous immunoglobulin** (IVIg; a treatment made of purified antibodies from donated adult blood) or **plasma exchange** (a procedure to remove some antibodies from the pregnant individual's blood) to delay the development of anemia and the need for IUTs, but there is limited evidence on how well these approaches work

More information about HDFN management in clinical settings, along with outcomes for pregnant individuals, their children, and their families following an HDFN-affected pregnancy, is essential for improving the outcomes of HDFN.

The GERANIUM registry is a global, prospective, non-interventional study that will collect real-world data from pregnant participants at risk for HDFN, as well as their babies/children.

STUDY SITES ARE IN ~21 SPECIALIZED CENTERS

Located in **North America, Latin America, Europe, and Asia Pacific**

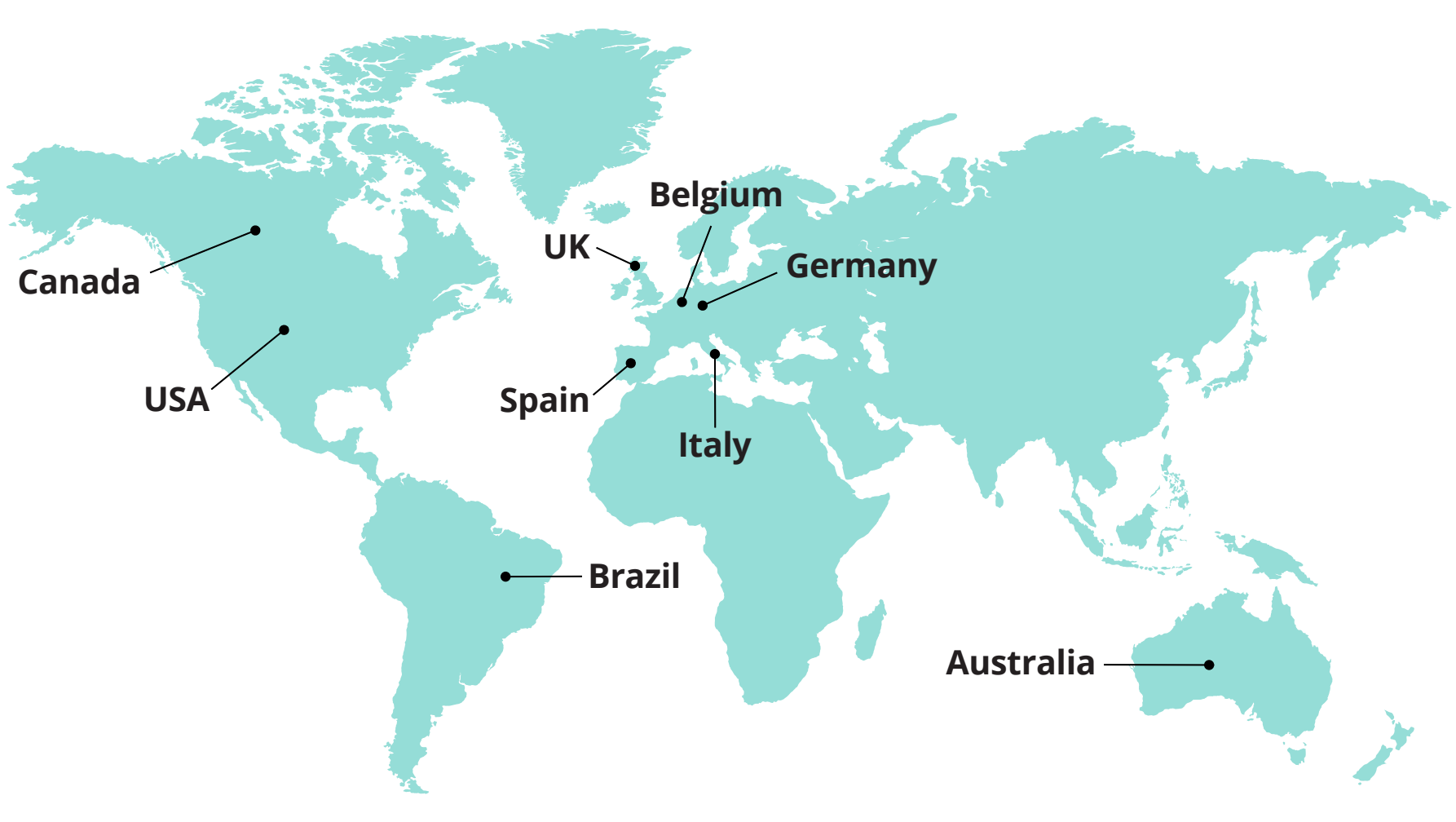

The GERANIUM registry is not a clinical trial, meaning it does not test new treatments. Instead, **it collects real-world information from routine medical care** to understand how HDFN is managed.

Study population

Pregnant individuals aged ≥18 years with:

- Gestational age up to 24 weeks
- History of HDFN in ≥1 prior pregnancy
- Current pregnancy with the presence of maternal alloantibodies and an antigen-positive fetus

Study design

- Maternal follow-up will occur during the pregnancy and for up to 2 years after delivery
- Follow-up for babies/children will occur from birth until they are up to 2 years old
- Data from pregnant participants and their children will be linked to provide a more comprehensive understanding of how HDFN affects families throughout pregnancy and in the early years of infancy/childhood

GERANIUM will collect information **prospectively**, meaning the researchers plan to study outcomes that have not yet happened. They will use **medical records, optional questionnaires, and optional blood tests** to gather data.

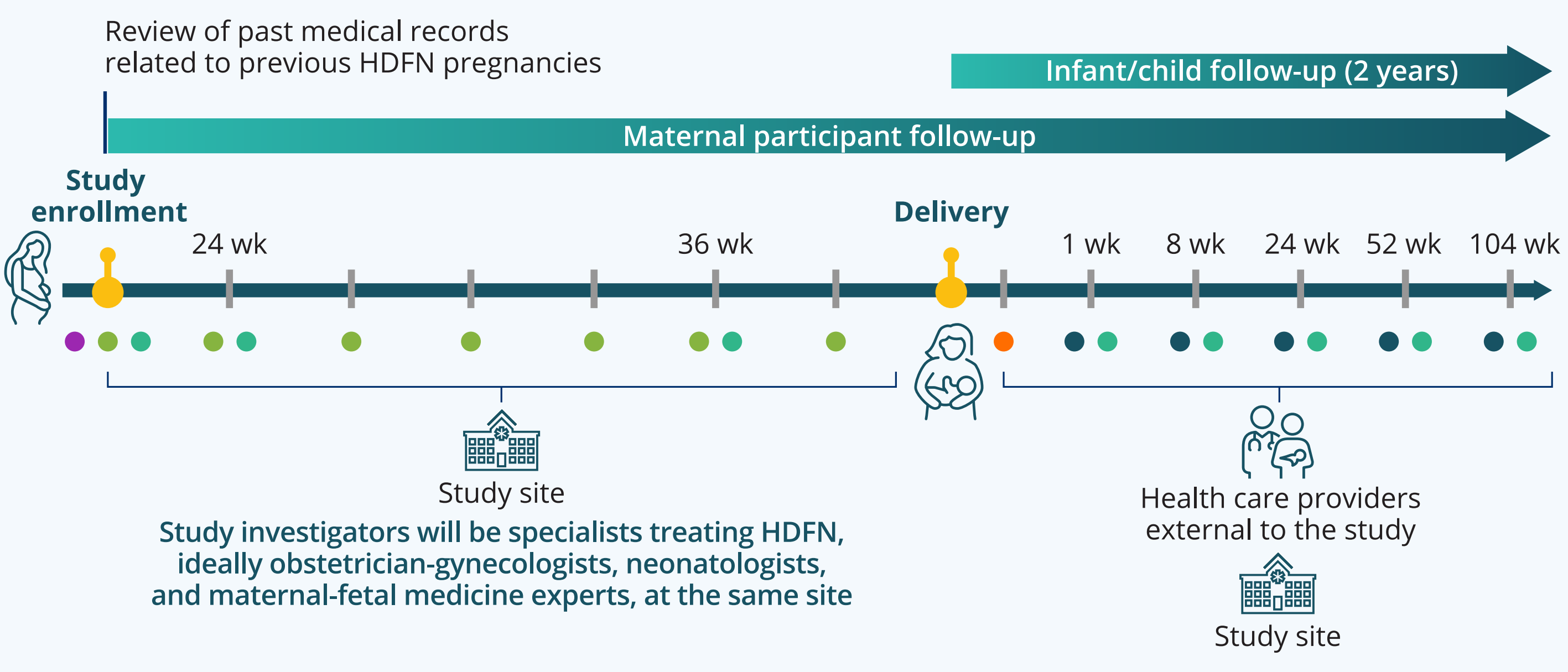

Data collected in the GERANIUM registry will be analyzed to understand a range of outcomes in HDFN-affected pregnancies.

Primary endpoint:

- Number of pregnancies that continue without the loss of a fetus/baby, need for IUT, or development of fetal hydrops

Key secondary endpoints:

- Severity of HDFN, as measured by a composite HDFN severity index
- Length of time before the first IUT or the development of fetal hydrops
- The modified Neonatal Mortality and Morbidity Index (mNMMI) in liveborn neonates
- Number of IUTs received during pregnancy

Birth/postnatal outcomes:

- Gestational age at delivery
- Length of stay in the neonatal intensive care unit
- Number of babies receiving transfusions/IVIg
- Number of babies treated with phototherapy

Longer-term developmental outcomes:

- Growth and development (length/height, weight, and head circumference)
- Number of children with abnormal growth, development, or hearing
- Number of children with neurodevelopmental, behavioral, or dental disorders

Other assessments:

- Questionnaires from participants/partners about their health and well-being, as well as that of their children
- Treatment pathways associated with HDFN diagnosis
- Use of medical services like hospitalizations or doctor's visits
- Assessments of risk factors for the development of HDFN and responsiveness to treatment

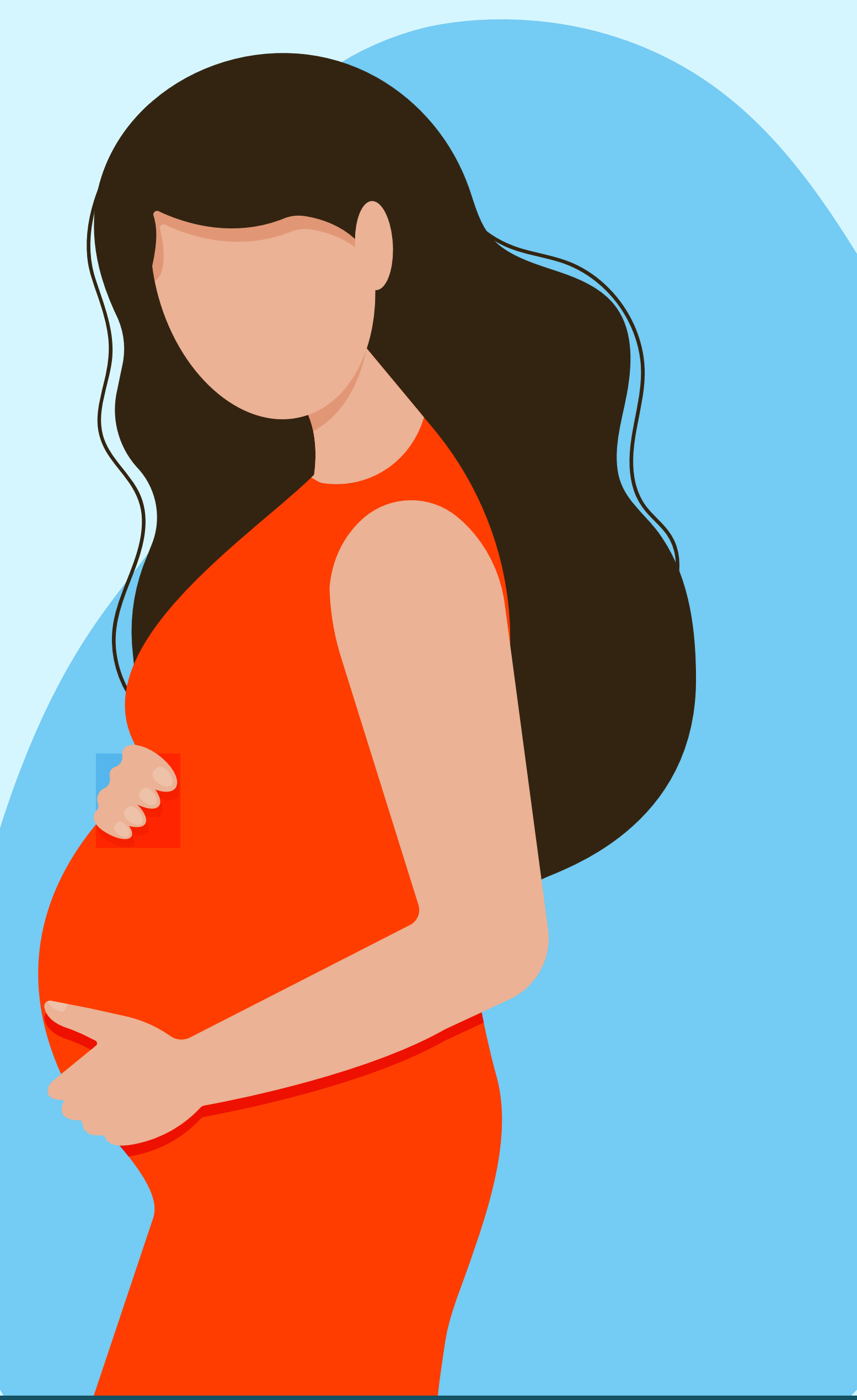

The GERANIUM registry will provide new information about how HDFN is treated in everyday clinical practice across different settings.

Findings from this registry may help researchers and doctors find ways to improve patient care, help shape future research, and provide support to families affected by HDFN worldwide.
